# Metabolomics-Based Analysis of Geographical Origin-Driven Quality Variation in Cultivated *Pyropia haitanensis*

**DOI:** 10.3390/foods15081299

**Published:** 2026-04-09

**Authors:** Wenjing Zhu, Kai Xu, Yan Xu, Dehua Ji, Wenlei Wang, Chaotian Xie

**Affiliations:** 1 Fisheries College, Jimei University, Xiamen 361021, China; 2 Fujian Engineering Research Center of Aquatic Breeding and Healthy Aquaculture, Xiamen 361021, China; 3 State Key Laboratory of Mariculture Breeding, Fisheries College of Jimei University, Ningde 352121, China

**Keywords:** *Pyropia haitanensis*, geographical origin, quality differences, metabolomics, chemometrics

## Abstract

*Pyropia haitanensis*, an economically significant cultivated seaweed in China, exhibits substantial geographical variations in nutritional and sensory qualities that influence its market value. The nutritional quality of the samples, including total sugar, total protein, and amino acid content, as well as color quality, assessed through phycobiliprotein and chlorophyll content, and sensory quality evaluated using an electronic nose and electronic tongue, were determined. To elucidate these quality variations, this study employed an integrated metabolomics and chemometrics approach to analyze samples from five major cultivation regions. Principal component analysis (PCA) effectively differentiated the samples; orthogonal partial least squares discriminant analysis (OPLS-DA) validated this classification with robust model parameters (R^2^X = 0.791, R^2^Y = 0.995, Q^2^ = 0.984) and identified key discriminatory metabolites. Weighted gene co-expression network analysis (WGCNA) identified origin-specific metabolic modules correlated with quality traits, revealing that pathways such as cysteine and methionine metabolism underpin the observed differences in flavor profiles across cultivation regions. Furthermore, mediation analysis quantitatively confirmed that inorganic nitrogen primarily influences key flavor attributes by regulating sulfur-containing amino acid and nucleotide metabolism. This study systematically elucidates the metabolic mechanisms governing quality formation in *P. haitanensis*, providing a scientific foundation for quality control and geographical origin traceability.

## 1. Introduction

Species of the genera *Pyropia*/*Porphyra* are economically significant seaweeds with a cultivation history spanning centuries in Asia [1]. These macroalgae are not only rich sources of proteins, amino acids, and minerals but also contain substantial quantities of bioactive compounds, including porphyran, vitamin B12, and taurine [2]. Phycobiliproteins derived from red algae and cyanobacteria have extensive applications as food colorants, cosmetic ingredients, and fluorescent markers in biomedical research [3]. Porphyran, a characteristic sulfated polysaccharide in Pyropia/Porphyra, exhibits diverse bioactivities, including anti-cancer [4], antioxidant [5], and anti-inflammatory [6] properties. Vitamin B12, abundant in these species [7], is essential for physiological processes such as nervous system function and erythropoiesis [8]. Taurine is one of the major sulfonic acid derivatives in *Pyropia*/*Porphyra* species and other red algae, with possible uses in several disease treatments [9]. Traditionally, geographical origin traceability underscores the value of Pyropia/Porphyra species as highly nutritious potential functional foods. Furthermore, commercial cultivation of these species contributes to global sustainability targets due to their low carbon footprint, high nutrient density, and positive ecological role [10].

*Pyropia haitanensis*, which stands out as a dominant mariculture species in China [11], is widely cultivated with the highest annual production among all *Pyropia*/*Porphyra* species [12]. China produces approximately 88,000 tons (dry weight) of *P. haitanensis* annually [13], accounting for over 70% of the total production in China [14]. Cultivation is primarily concentrated in the coastal regions of Fujian, Zhejiang, and Guangdong provinces [15], with recent expansion into Jiangsu and Shandong provinces [16]. The escalating market demand for *P. haitanensis* has been accompanied by a growing consumer preference for high-quality thalli, which command a price premium driven largely by superior sensory attributes, particularly flavor. However, environmental factors and cultivation practices can cause significant regional variations in key quality characteristics, such as nutrient composition, texture, and flavor profiles [17]. For example, the brown alga Saccharina latissima used in Japanese cuisine, harvested from different coastal sites exhibits significant geographical variation in protein, carbohydrate, and mineral contents [18], while comparative analysis of *Gracilaria multipartita*, *Sargassum muticum* and *Ulva* sp. from distinct locations revealed location-specific elemental and nutritional profiles [17]. Similarly, the polar lipid profiles of commercial *Ulva* spp. vary significantly with geographical origin, influencing their nutritional and functional value for commercial applications [19]. *Kappaphycus alvarezii* from 10 Indonesian farming locations demonstrates highly variable production and carrageenan quality (e.g., gel strength, viscosity) [20]. In *P. haitanensis* specifically, nitrogen and phosphorus availability directly affects pigment accumulation, amino acid metabolism, and taste-related compounds [21], establishing the mechanistic basis for the regional variations in nutrient composition, texture, and flavor profiles observed across Chinese cultivation sites. Systematic metabolic fingerprinting of these geographical effects, particularly regarding quantitative linkages between environmental gradients, metabolic profiles, and sensory quality, remains underexplored. Distinguishing these quality differences visually is challenging, as processed thalli from diverse origins often exhibit remarkable similarity in morphology and color. Traditionally, geographical origin traceability and quality assessment have relied on subjective sensory evaluations of appearance and taste [22]. However, these approaches lack the precision and reproducibility required for modern food safety and quality control standards and they fail to establish quantitative linkages between metabolic profiles and sensory attributes, necessitating the development of rapid, accurate, and objective analytical methods based on metabolomic fingerprints.

Electronic nose (E-nose) and electronic tongue (E-tongue) technologies have been introduced as cost-effective, objective alternatives to human sensory evaluation, capable of assessing holistic food quality through volatile and taste profiling [23]. While these instrumental methods effectively bridge the gap between chemical analysis and sensory perception, overcoming the subjectivity of human panels, they primarily provide phenotypic discrimination rather than mechanistic insight. To decipher the underlying biochemical mechanisms of food quality, metabolomics has emerged as a pivotal tool. Metabolomics offers a comprehensive characterization of chemical components, enabling the simultaneous identification and quantification of thousands of metabolites [24]. It has proven effective in identifying chemical markers associated with quality traits in crops such as dragon fruit [25] and *Polygonatum cyrtonema* [26]. Recent studies have further demonstrated its utility; for instance, Ye et al. [27] utilized sensory-metabolomics to elucidate the metabolic basis of quality changes in jujube during storage, while He et al. [28] combined molecular sensory science with metabolomics to screen key aroma-contributing compounds in fermented Baijiu. These findings underscore the capacity of metabolomics to identify metabolic signatures and pathway associations that discriminate quality variations, providing a foundation for biosynthetic hypotheses. Among available metabolomic platforms, NMR requires large sample amounts and GC-MS is limited to volatile/derivatized compounds [29], whereas UPLC-MS/MS, which offers high sensitivity, strong specificity, short analysis time, and good reproducibility, has been successfully applied to metabolomics studies of *P. haitanensis* [16].

This study aimed to identify origin-discriminatory metabolites and link metabolites to sensory traits in *P. haitanensis* from five major production areas. Using UPLC-MS/MS-based metabolomics combined with chemometric and sensory analyses, this work provides a metabolic fingerprinting approach for objective geographical origin discrimination and quality evaluation of *P. haitanensis*.

## 2. Materials and Methods

### 2.1. Sample Collection and Environmental Monitoring

**Figure 1:**
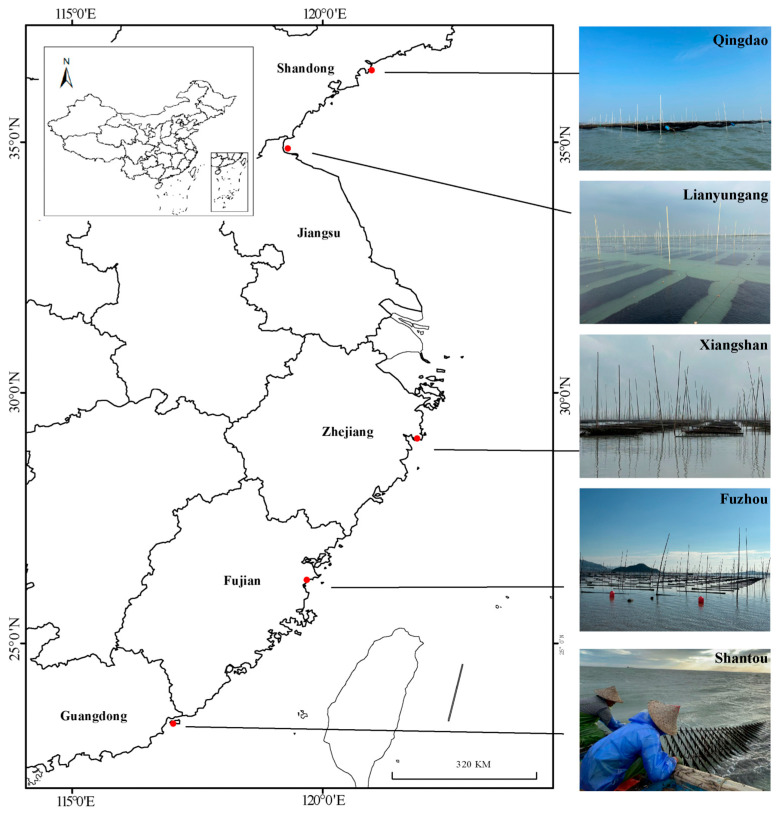
Geographic distribution map of sample collection sites for *Pyropia haitanensis* in five coastal provinces of China (Shandong, Jiangsu, Zhejiang, Fujian, and Guangdong).

From September to November 2023, samples were collected from the main *P. haitanensis*-producing areas in Qingdao City, Shandong Province (QD, N 36.43182733°, E 120.9763392°), Lianyungang City, Jiangsu Province (LYG, N 34.8761645°, E 119.2995412°), Xiangshan City, Zhejiang Province (NB, N 29.08717283°, E 121.8832872°), Fuzhou City, Fujian Province (FZ, N 26.2702575°, E 119.68292267°), and Shantou City, Guangdong Province (ST, N 23.4067035°, E 117.01233617°), in China (Figure 1). To ensure reproducibility and minimize environmental variability, all samples were collected during the first harvest (first cutting) of the cultivation season. For each geographical region, four biological replicates were obtained from different cultivation racks. Each replicate consisted of over 100 individual thalli, which were pooled, shade-dried at room temperature, ground into a homogeneous powder, and stored at −20 °C until analysis. For metabolomics analysis, shade-dried samples were subsequently freeze-dried to ensure complete dehydration and optimal metabolite extraction efficiency.

Concurrently, environmental factors were measured following previously established protocols at each biological replicate site (cultivation rack) [30]. At each location, measurements were performed once at each of the three biological replicate sites. The monitored parameters included water temperature (WT), salinity (Sal), transparency (Trans), dissolved oxygen (DO), pH, total nitrogen (TN), inorganic nitrogen (IN), total phosphorus (TP), total organic carbon (TOC), total carbon (TC), and inorganic carbon (IC). Specifically, salinity was determined using a hand-held refractometer (Suwei, Guangzhou, China) and transparency was assessed via the Secchi disk method. WT, DO, and pH were measured in situ using a multi-parameter meter (Oxi 340i; WTW, Weilheim, Germany). Concentrations of TOC, IC, and TC were quantified using a total organic carbon analyzer (TOC-5000A; Shimadzu, Kyoto, Japan), while TP, TN, and IN levels were analyzed using a CleverCHEM 380 automated analyzer (DeChem-Tech GmbH, Tübingen, Germany).

### 2.2. Determination of Total Sugar, Protein, and Phycobiliprotein Contents in P. haitanensis

The total sugar content was determined according to a published method [31], by measuring the absorbance at 562 nm. Total protein content was determined using a BCA Protein Assay Kit (Solarbio, Beijing, China) by measuring the absorbance at 540 nm. Absorbance for both assays was measured using a microplate reader (Multiskan GO; Thermo Fisher Scientific, Waltham, MA, USA). For total amino acid analysis, protein hydrolysis was performed with 6 M HCl at 110 °C for 22 h prior to detection using an automated amino acid analyzer (LA8080; Hitachi, Tokyo, Japan) [32]. Chlorophyll was extracted with 90% acetone at 4 °C for 24 h in the dark, and the content was determined by measuring the absorbance at 666 nm and 730 nm. Phycobiliprotein was extracted with 0.05 M PBS (pH 6.8) at 4 °C for 48 h in the dark, and the content was determined by measuring the absorbance at 565 nm, 615 nm, 650 nm, and 730 nm using a spectrophotometer (UV-1900i; Shimadzu, Kyoto, Japan) [33].

### 2.3. Metabolite Extraction and LC-MS/MS Analysis

Widely targeted metabolomic profiling was performed by Wuhan Metware Biotechnology Co., Ltd. (Wuhan, China) based on established protocols [34]. For metabolite extraction, freeze-dried samples were ground to powder and 50 mg was extracted with 1200 μL pre-cooled (−20 °C) 70% methanol aqueous solution containing 2-chloro-L-phenylalanine (1 ppm) as an internal standard. After vortexing (6 cycles, 30 s each) and centrifugation (12,000 rpm, 3 min), the supernatant was filtered (0.22 μm) for analysis. QC samples (pooled extracts) were inserted every 10 samples to monitor reproducibility.

Chromatographic separation was performed on an ExionLC AD (https://sciex.com.cn/) UPLC system with an Agilent SB-C18 column (1.8 μm, 2.1 mm × 100 mm). The mobile phase was (A) ultrapure water with 0.1% formic acid and (B) acetonitrile with 0.1% formic acid. Gradient elution: 0–9 min, 5–95% B; 9–10 min, 95% B; 10–11.1 min, 95–5% B; equilibration to 14 min. Flow rate: 0.35 mL/min; column temperature: 40 °C; injection volume: 2 μL.

Mass spectrometry used a triple quadrupole mass spectrometer in MRM mode with ESI source temperature 550 °C, ion spray voltage 5500 V (+)/−4500 V (−), and GSI/GSII/CUR gases at 50/60/25 psi.

Data processing: Missing values were imputed with 1/5 of the minimum value per metabolite. Metabolites with QC CV > 0.5 were excluded. Peak areas were integrated using MultiQuant software (version 3.0.3, AB Sciex) for relative quantification. Metabolite identification matched retention times, precursor ions (Q1) and product ions (Q3) against the Metware database (MWDB). The detailed MS1 and MS2 information for target metabolites is provided in Supplementary Table S1.

### 2.4. Electronic Nose Analysis

Volatile profile analysis was conducted using a PEN3 electronic nose (Airsense Analytics GmbH, Schwerin, Germany) following the protocol of Zhou et al. [35]. Briefly, 5.0 g of sample was placed in a 20 mL headspace vial and incubated in a 50 °C water bath for 30 min to equilibrate volatiles. The E-nose probe was then inserted to sample the headspace gas. The instrument parameters were set as follows: sampling interval, 1 s; sensor cleaning time, 200 s; zeroing time, 10 s; sample preparation time, 5 s; and sample/sensor flow rates, 400 mL/min. Data acquisition lasted 120 s, with the stable signal period from 115 s to 118 s selected for analysis. The sensor array consisted of 10 metal oxide semiconductor sensors (W1C, W5S, W3C, W6S, W5C, W1S, W1W, W2S, W2W, and W3S).

### 2.5. Electronic Tongue Analysis

Taste attributes were evaluated using an SA402B electronic tongue (Intelligent Sensor Technology, Inc., Atsugi, Japan) according to Yuan et al. [13]. A 1.0 g sample was mixed with 50 mL of deionized water and extracted at room temperature for 30 min with stirring. The supernatant obtained after filtration served as the test solution. Prior to measurement, the lipid/polymer membrane sensors were preconditioned, calibrated, and checked for stability to ensure data accuracy.

### 2.6. Color Determination

The color of *P. haitanensis* thalli was measured using a portable colorimeter (CHROMA METER CR-400, Konica Minolta, Tokyo, Japan) under standard D65 daylight illumination (color temperature 6500 K). The instrument was calibrated with the provided standard white plate prior to measurements. For each sample, the parameters of the CIE color space, L* (light), a* (red-green), and b* (yellow-blue) values, were recorded at three positions: apical, middle, and basal portions of the thallus. Three individual thalli were measured per location, and all values were averaged for statistical analysis.

### 2.7. Statistical Analysis

Data visualization was performed using GraphPad Prism (version 8.3.0; GraphPad Software, San Diego, CA, USA). Multivariate statistical analysis, including Orthogonal Partial Least Squares-Discriminant Analysis (OPLS-DA), was conducted using SIMCA 14.1 (Umetrics, Umeå, Sweden). Prior to OPLS-DA modeling, raw data underwent preprocessing with a missing value tolerance of 50% for both variables and observations; missing values were imputed using median values. Both X-block (metabolite matrix) and Y-block (class membership) were scaled using unit variance (UV) scaling to normalize variable contributions. Metabolic pathway analysis and enrichment were performed using the MetaboAnalyst platform (www.metaboanalyst.ca) to identify key pathways associated with differentially abundant metabolites.

## 3. Results

### 3.1. Determination of Quality-Related Traits for P. haitanensis from Different Regions

**Figure 2:**
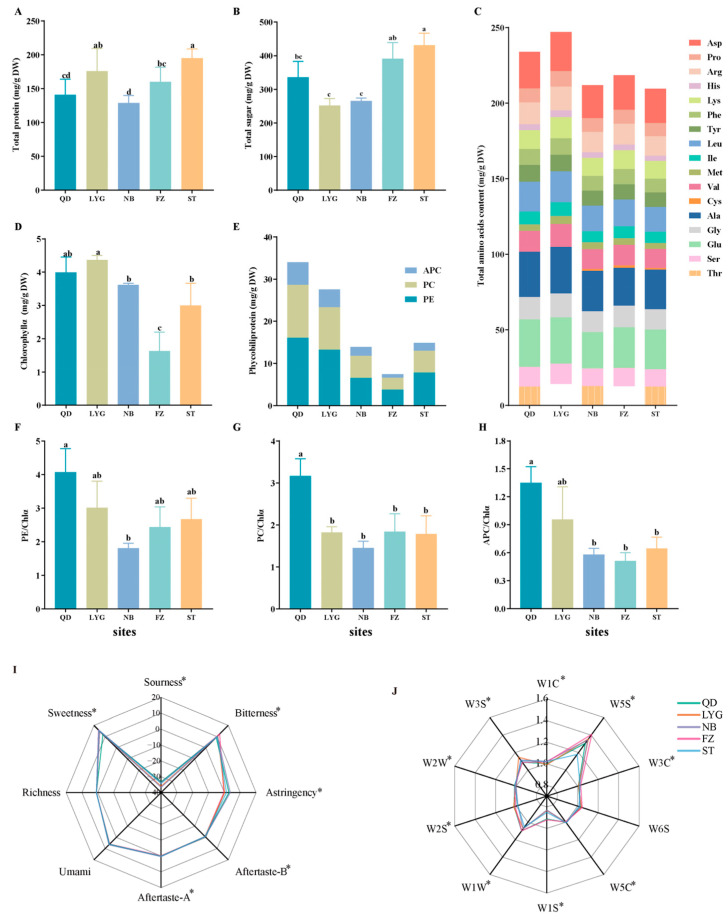
Quality-related indices of *Pyropia haitanensis* from five major mariculture regions in China. (**A**) Total protein content. (**B**) Total sugar content. (**C**) Contents of 17 hydrolyzed amino acids. (**D**) Chl *a* content. (**E**) Phycobiliprotein content. (**F**) PE/Chl *a*. (**G**) PC/Chl *a*. (**H**) APC/Chl *a*. (**I**) Electronic tongue taste profile radar chart. (**J**) Electronic nose olfactory profile radar chart. Chl *a*: chlorophyll *a*; APC: allophycocyanin; PC: phycocyanin; PE: phycoerythrin. Aftertaste-A (bitterness aftertaste) and Aftertaste-B (astringency aftertaste) represent the residual intensity of bitterness and astringency after swallowing, respectively, as detected by the electronic tongue sensor array. Different lowercase letters above bars in subfigures A, B, D, E, F, G and H indicate significant differences among groups (*p* < 0.05, Tukey’s test). Asterisks (*) in subfigures I and J indicate significant differences between groups (*p* < 0.05).

The nutritional quality of *P. haitanensis*, characterized by its protein and sugar composition, exhibited significant geographical variation. Total protein content ranged from a minimum of 130 mg/g in Ningbo (NB) samples to a maximum of approximately 200 mg/g in Shantou (ST) samples (Figure 2A). Interestingly, total soluble sugar content followed a distinct trend; ST samples contained the highest levels (430 mg/g), whereas Lianyungang (LYG) samples exhibited significantly lower contents (250 mg/g) compared to other regions (Figure 2B). Amino acid composition is a fundamental determinant of both nutritional value and flavor potential. A total of 17 amino acids were quantified in the hydrolyzed samples. Glutamic acid (Glu) and aspartic acid (Asp) were identified as the predominant components across all samples. Total amino acid content showed significant regional heterogeneity, peaking in LYG samples while remaining lowest in ST samples (Figure 2C). Notably, the levels of Asp, Glu, Ala, and Gly differed significantly among origins (*p* < 0.01). Qingdao (QD) samples exhibited the highest Glu concentrations, whereas NB samples showed the lowest levels of Glu, Asp, and Phe. These distinct amino acid profiles reflect regional variations in the nutritional quality of thalli from different cultivation regions.

*Pyropia* thallus color reportedly changes based on environmental conditions [36]. Pigment types and relative contents as well as their ratios are the main factors influencing thallus coloration [37]. Our analysis revealed significant regional differences in pigment profiles. Fuzhou (FZ) samples exhibited the lowest contents of both chlorophyll *a* (Chl *a*) and phycobiliproteins. In contrast, phycobiliprotein levels were highest in QD samples, while Chl *a* content peaked in LYG samples (Figure 2D). Furthermore, the proportions of phycobiliproteins and their ratios to Chl *a* (PE/Chl *a*, PC/Chl *a*, and APC/Chl *a*) varied significantly among locations (*p* < 0.05; Figure 2E–H). Colorimetric analysis revealed significant regional variation in thallus color parameters. L* (lightness) values differed significantly among populations (*p* < 0.05), with samples from LYG exhibiting the highest lightness and those from FZ the lowest (Figure S1A). Similarly, a* (green–red) values varied significantly across regions (*p* < 0.05): LYG and ST samples displayed positive a* values (indicating a reddish hue), whereas QD, NB, and FZ samples exhibited negative a* values (indicating a greenish hue) (Figure S1B). A scatter plot of samples in L*a* space—with point size scaled to represent b* values—showed clustering by geographic origin (Figure S1C), providing visual confirmation of pronounced regional differences in thallus coloration. Correlation analysis revealed a significant positive association between L* and Chl *a* content (r = 0.59, *p* < 0.05; Figure S1D), confirming that chlorophyll is the primary determinant of thallus lightness in *P. haitanensis*. These variations in pigment stoichiometry provide a biochemical explanation for the distinct visual characteristics observed in *P. haitanensis* from different origins.

### 3.2. Sensory Evaluation

The taste profiles of *P. haitanensis* from different geographical origins were characterized using an electronic tongue (Figure 2I). The radar chart analysis revealed that the sweetness sensor elicited the strongest electrical response across all samples, followed by the umami sensor. In contrast, response values for the sourness sensor were negligible (below 0), suggesting minimal acidic detection. These sensor response patterns suggest that sweetness and umami may contribute prominently to the electronic tongue-detectable taste profile of *P. haitanensis*.

Volatile detection was performed using an electronic nose equipped with 10 metal oxide semiconductor (MOS) sensors. Strong electrical responses were observed for sensors W5S (manufacturer-specified sensitivity: nitrogen oxides), W1W (sulfides and pyrazines) and W3S (alkanes), indicating that these sensors underwent significant conductivity changes upon volatile exposure. While the overall radar chart patterns (Figure 2J) suggested qualitative similarity in sensor response profiles across different cultivation regions, significant quantitative variations were evident in response magnitudes and relative sensor signal proportions, indicating that the intensity of volatile profiles varies substantially by geographical origin.

### 3.3. Analysis of Differences in Metabolites Among P. haitanensis Samples from Different Geographical Regions

**Figure 3:**
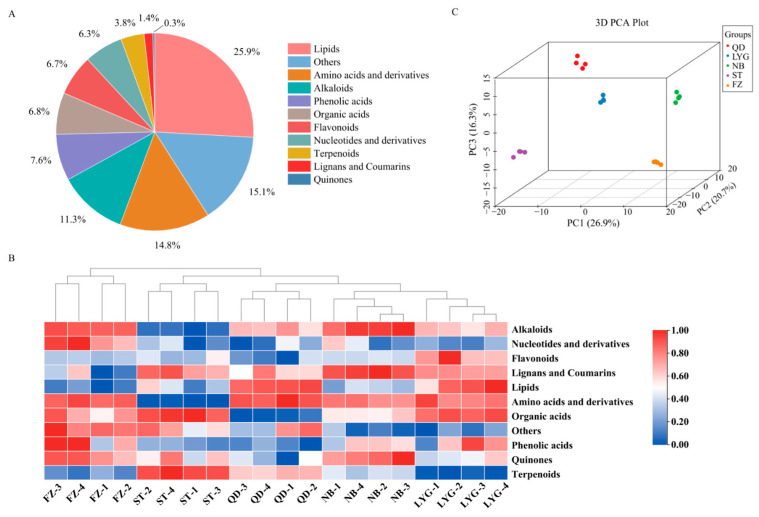
Comprehensive geographical metabolomic profiling of *Pyropia haitanensis* across five major mariculture regions in China. (**A**) Pie chart of metabolite abundance. (**B**) Hierarchical clustering of different *P. haitanensis*-producing areas and correlations between metabolites and *P. haitanensis* geographical origins. (**C**) Unsupervised PCA classification of *P. haitanensis* samples.

A widely targeted metabolomics approach identified a total of 630 metabolites classified into 11 categories, comprising 163 lipids, 93 amino acids and derivatives, 71 alkaloids, 48 phenolic acids, 43 organic acids, 42 flavonoids, 40 nucleotides and derivatives, 24 terpenoids, nine lignans and coumarins, two quinones, and 95 others (Figure 3A). Lipids represented the most abundant class, followed by amino acids and their derivatives, while quinones were the least prevalent. This coverage significantly exceeds that of previous metabolomic studies on *P. haitanensis*, which typically reported between 218 [13] and 597 [38] metabolites, with the expanded coverage primarily observed in lipids, amino acids, and alkaloids. The observed metabolic diversity among samples was predominantly quantitative, likely reflecting the combined influence of genotype and environmental factors [39]. These systematic variations in metabolite abundance provide a molecular framework for understanding the divergence in nutritional and sensory qualities observed across different cultivation regions.

To evaluate the overall metabolic differences among origins, multivariate statistical analyses were performed. Hierarchical clustering analysis (HCA), visualized as a heatmap (Figure 3B), demonstrated that samples from the same cultivation region formed distinct, tightly correlated clusters, indicating high intra-group consistency. Unsupervised principal component analysis (PCA) further corroborated these findings by revealing the intrinsic structure of the dataset (Figure 3C). The 3D PCA score plot incorporating the first three principal components (PC1 = 26.9%, PC2 = 20.8%, and PC3 = 16.3%; cumulatively 64.0%) showed clear spatial separation among the five geographical groups, with biological replicates clustering closely, confirming high data reproducibility. The scree plot (Figure S2) exhibited a pronounced elbow at PC4, where variance dropped sharply from 15.2% to 2.8%, confirming that the first three components captured the majority of meaningful variation. These results compellingly demonstrate that geographical origin is a primary driver of systematic metabolic differentiation in *P. haitanensis*. Such distinct metabolic profiles are likely responsible for the regional variations in nutritional and sensory attributes discussed previously.

### 3.4. OPLS-DA Analyses of P. haitanensis Geographical Origins

**Figure 4:**
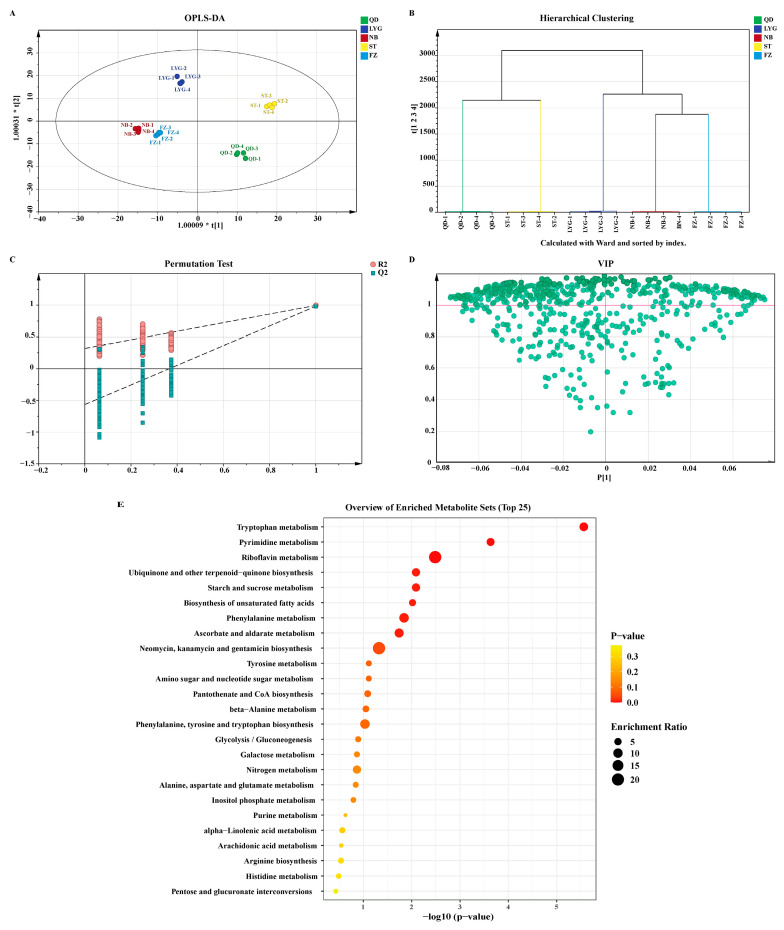
OPLS-DA model of *Pyropia. haitanensis* from different geographical regions. (**A**) OPLS-DA score chart. (**B**) Hierarchical clustering chart based on Ward. (**C**) Results of 200 permutation tests of the OPLS-DA model. (**D**) VIP values for the OPLS-DA model. (**E**) KEGG pathway enrichment analysis of metabolites with VIP > 1.

To deepen the understanding of regional metabolic disparities, orthogonal partial least squares-discriminant analysis (OPLS-DA) was performed using SIMCA 14.1 with UV scaling. Hotelling’s T2 test confirmed the absence of outliers, with all samples falling within the 95% confidence interval [40]. The constructed model exhibited excellent explanatory and predictive capabilities, characterized by high goodness-of-fit (R^2^X = 0.791, R^2^Y = 0.995) and predictive ability (Q^2^ = 0.984) [41]. The score plot (Figure 4A) revealed distinct separation among geographical groups with tight intra-group clustering, demonstrating the model’s efficacy in discriminating *P. haitanensis* origins. This classification was further validated by hierarchical clustering analysis based on Ward’s method (Figure 4B), which yielded consistent results. Model robustness was verified via 200 permutation tests. The results showed that all permuted R^2^ and Q^2^ values were lower than the original model, with intercepts of R^2^ = (0.0, 0.328) and Q^2^ = (0.0, −0.591). The negative intercept of the Q^2^ regression line confirmed the absence of overfitting. (Figure 4C) [42]. To identify key discriminatory metabolites, Variable Importance in Projection (VIP) scores were utilized [43]. A total of 414 metabolites, representing 65.7% of the identified compounds, possessed VIP values > 1 (Figure 4D). These metabolites, which also exhibited significant differential abundance (*p* < 0.05), serve as promising biomarkers for elucidating the mechanisms underlying geographical quality variation. The model achieved perfect classification accuracy with AUC = 1.0 for all five geographical origins.

Performing pathway enrichment analysis on these metabolites revealed that they were significantly enriched in multiple core metabolic pathways (Figure 4E). Notably, these pathways can be broadly categorized into three key functional groups: (1) Amino Acid and Nitrogen Metabolism: Several pathways directly involved in amino acid biosynthesis and metabolism were prominently enriched, viz. alanine, aspartate and glutamate metabolism; arginine biosynthesis; phenylalanine, tyrosine and tryptophan biosynthesis; and nitrogen metabolism. The enrichment of these pathways provides a direct metabolic basis for the observed regional disparities in total protein content and the profiles of umami and bitter amino acids (as shown in Figure 2A,C). (2) Energy and Carbohydrate Metabolism: pathways such as glycolysis/gluconeogenesis, starch and sucrose metabolism, and galactose metabolism were significantly altered. This indicates that fundamental energy homeostasis and carbon skeleton provision differ among *P. haitanensis* from different environments, which could underpin variations in total sugar content (Figure 2B) and overall growth dynamics. (3) Lipid and Cofactor Biosynthesis: The enrichment of biosynthesis of unsaturated fatty acids, alpha-linolenic acid metabolism, and ubiquinone and other terpenoid-quinone biosynthesis points to geographical differences in lipid composition and redox-related cofactors. These differences are likely to influence the nutritional quality of the thalli from different mariculture areas.

### 3.5. WGCNA Uncovers Origin-Specific Metabolic Modules Driving Quality Traits

**Figure 5:**
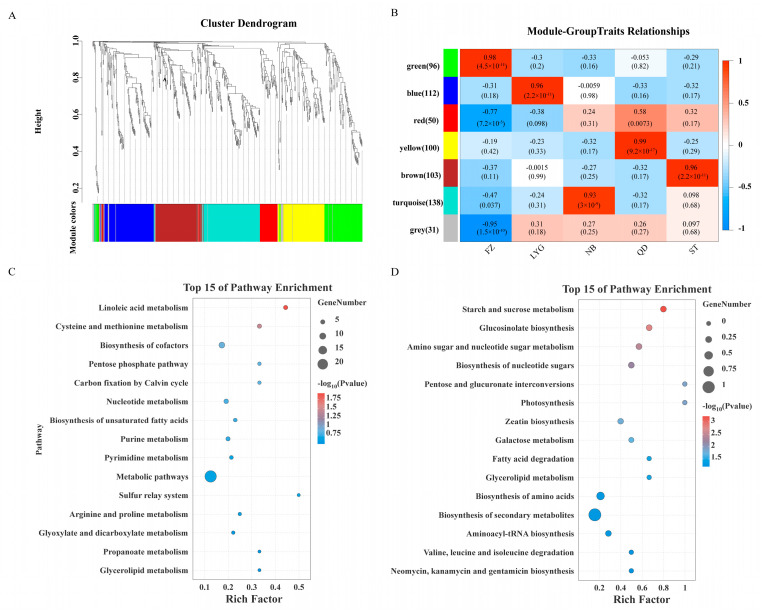
Weighted gene co-expression network analysis (WGCNA) of metabolites and identification of key modules. (**A**) Clustering dendrograms of metabolites and modules division. (**B**) Modules expression pattern analysis. (**C**) KEGG pathway enrichment of the yellow module. (**D**) KEGG pathway enrichment of the green module.

To systematically elucidate the metabolic basis of quality variations, a weighted gene co-expression network analysis (WGCNA) was performed on the 630 identified metabolites. This analysis clustered metabolites into seven co-expression modules (Figure 5A), five of which exhibited significant correlations with specific geographical origins. Fundamental pathways essential for primary metabolism, such as glycerolipid metabolism and the pentose phosphate pathway, were ubiquitously enriched across all modules (Figure S3), reflecting a conserved metabolic backbone in *P. haitanensis*. Superimposed on this conserved framework, origin-specific quality traits were defined by the differential enrichment of pathways within key modules.

The strong association between QD samples and the “yellow” module provides a metabolic explanation for their distinct flavor profile. Enrichment of “cysteine and methionine metabolism” within this module supplies precursors for sulfur-containing and aromatic volatiles (detected by sensors W1W and W2W; Figure 2J). Sulfur volatiles, despite their typically low abundance, are potent aroma contributors with low sensory thresholds and are key to heat-induced seafood flavors [44]. Furthermore, the pentose phosphate pathway generates precursors for aromatic amino acids [45], whose downstream derivatives likely contribute to the elevated aromatic compound signals (W2S, W2W) observed in these samples. Similarly, the correlation between FZ samples and the “green” module offers a metabolic rationale for their sensory characteristics. This module is enriched in bitter-tasting amino acids (e.g., isoleucine and leucine [46]), aligning with the elevated bitterness response recorded by the E-tongue (Figure 2I).

Mediation analysis was subsequently employed to statistically validate the causal pathways underlying these associations. Environmental factors exhibited significant regional heterogeneity (Figure S4), representing a nutrient-replete environment. We propose that these distinct environmental profiles create divergent growth regimes, which differentially modulate metabolic flux and stress response pathways in *P. haitanensis*. Consequently, this combination of differential environmental factors constitutes the primary external drivers shaping the geographical variations in metabolic composition and quality attributes.

Mediation analysis of the environment–metabolite–quality pathway revealed a significant negative indirect effect of IN on W1W sensor response via the sulfur-containing metabolite L-methionine sulfoxide (ACME = −0.153, *p* = 0.026), accounting for 89.5% of the total effect. This negative correlation indicates that lower inorganic nitrogen levels promote the accumulation of L-methionine sulfoxide, likely as a metabolic response to nitrogen-starvation-induced oxidative stress [47]. As an oxidation product of methionine, the accumulation of L-methionine sulfoxide under low-nitrogen conditions serves as a key metabolic signature that enhances the sulfurous flavor profile (W1W). Concurrently, a significant mediation path linking IN to W1W via the nucleotide metabolite 2-deoxyribose-1-phosphate was also identified (ACME = −0.116, *p* = 0.030; mediation proportion = 68.1%), suggesting a parallel contribution from nucleotide metabolism. Collectively, these findings demonstrate that environmental factors, particularly nitrogen availability, dictate final quality traits by regulating specific stress-responsive metabolic nodes.

## 4. Conclusions

This study systematically elucidated the geographical variation patterns in the quality and metabolic profiles of *P. haitanensis* across five major cultivation regions in China. Our findings revealed significant geographical heterogeneity in key nutritional components and sensory attributes. Chemometric models, including PCA and OPLS-DA, successfully discriminated the geographical origins of *P. haitanensis* based on metabolic fingerprints, with the OPLS-DA model achieving perfect classification performance (AUC = 1 for all five origins: SD, JS, ZJ, GD, and FJ). Notably, WGCNA identified specific metabolic modules closely associated with origin-specific quality traits. Furthermore, mediation analysis quantitatively confirmed that environmental inorganic nitrogen exerts a primary influence on the formation of characteristic flavors, particularly volatile sulfur compounds, by regulating key metabolites such as L-methionine sulfoxide. Collectively, this study provides a novel theoretical framework for the geographical traceability and quality control of *P. haitanensis*, offering mechanistic insights into the environmental regulation of flavor formation.

## Data Availability

The original contributions presented in this study are included in the article/Supplementary Materials. Further inquiries can be directed to the corresponding authors.

## Supplementary Materials

The following supporting information can be downloaded at https://www.mdpi.com/article/10.3390/foods15081299/s1: Figure S1. Regional variation in thallus color and its biochemical basis in *P. haitanensis*. (A,B) Regional differences in L* and a* values. (C) L*-a* distribution (point size = B). (D) Correlation between L* and Chl *a* content. Different lowercase letters in Figure S1 (A) and (B) indicate significant differences among groups (*p* < 0.05, Tukey’s test). Figure S2. Scree plot of principal components. Figure S3. KEGG enrichment analysis of gene co-expression modules. (A) KEGG pathway enrichment of the blue module. (B) KEGG pathway enrichment of the brown module. (C) KEGG pathway enrichment of the turquoise module. Figure S4. Environmental factors across sampling sites. (A) Water temperature (WT). (B) DO. (C) pH. (D) Sal. (E) Trans. (F) IN. (G) TN. (H) TP. (I) TOC. (J) TC. (K) IC. Different lowercase letters above bars indicate significant differences (*p* < 0.05) among sites (QD, LYG, NB, FZ, and ST). Table S1. Detailed MRM transition parameters (precursor ions, product ions) and retention times for key metabolites.

## Abbreviations

The following abbreviations are used in this manuscript:

PCA	Principal component analysis
OPLS-DA	Orthogonal partial least squares discriminant analysis
WGCNA	Weighted gene co-expression network analysis
E-nose	Electronic nose
E-tongue	Electronic tongue
