# Supplementary figures and images for: Metabolomics-Based Analysis of Geographical Origin-Driven Quality Variation in Cultivated Pyropia haitanensis
Source: Foods. 2026 Apr 9;15(8):1299. doi: 10.3390/foods15081299 (PMC13115154; doi:10.3390/foods15081299)

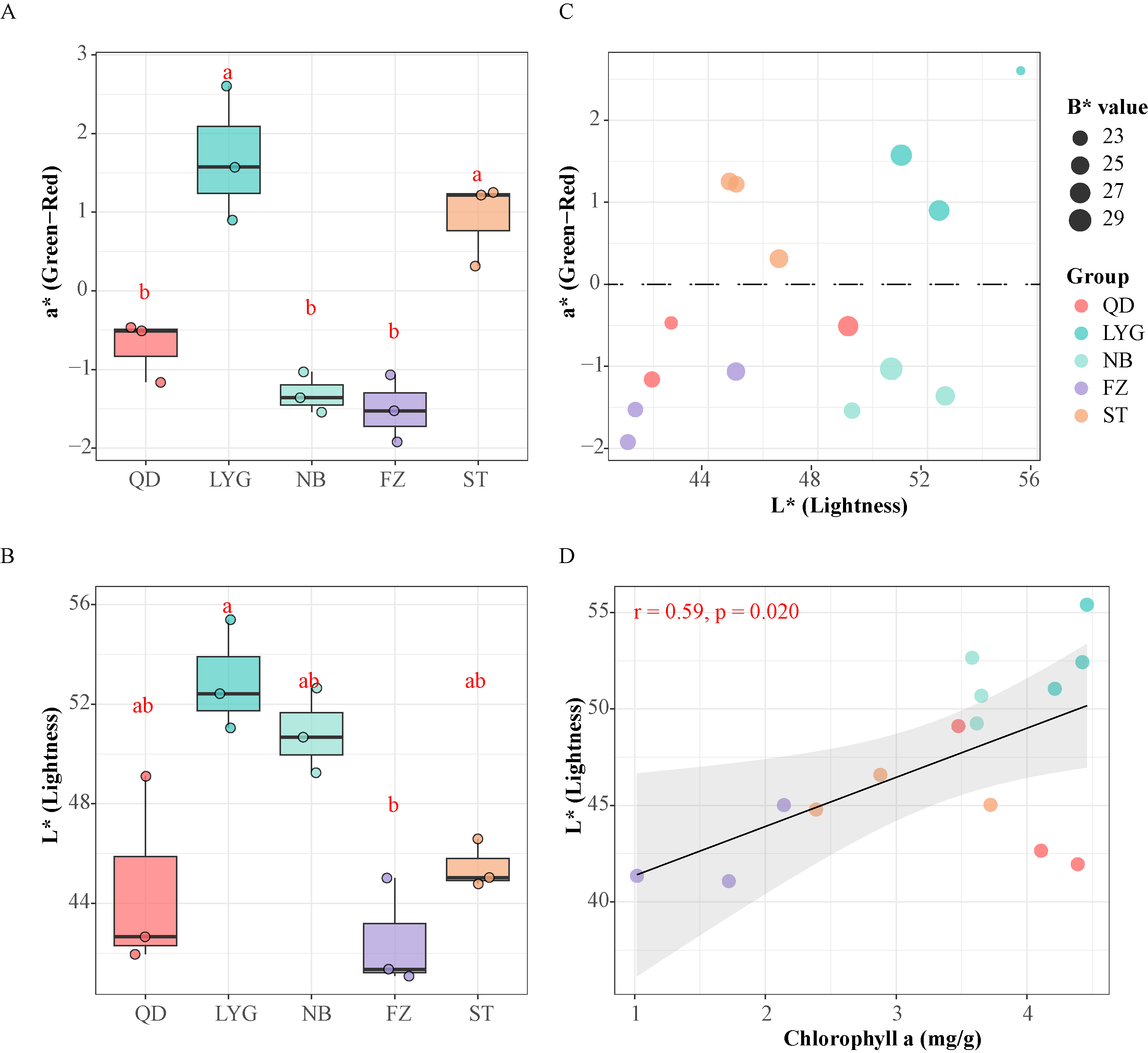

Supplement: Supplementary file 1 [file foods-15-01299-s001.zip › Figure S1.tif]

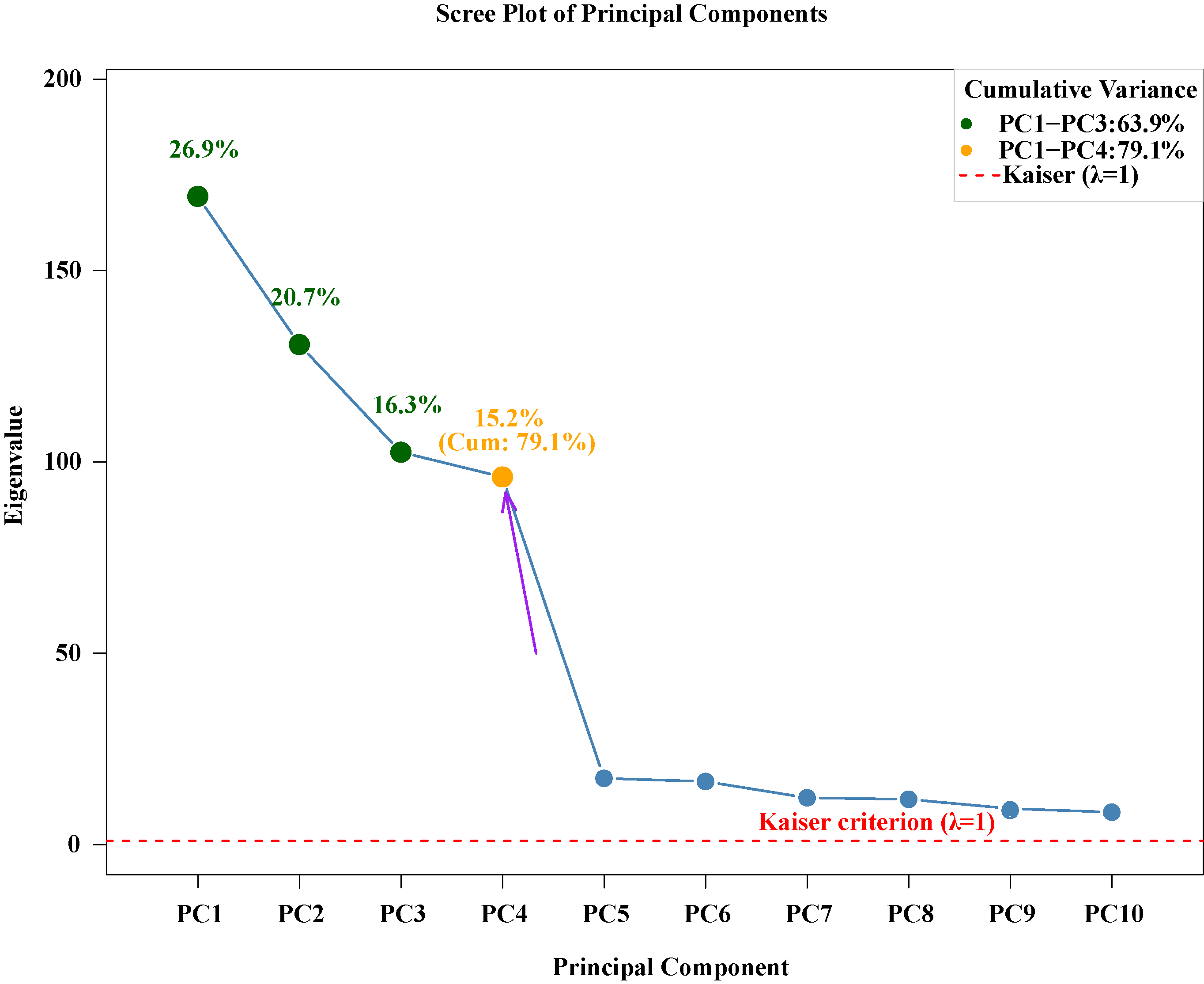

Supplement: Supplementary file 1 [file foods-15-01299-s001.zip › Figure S2.tif]

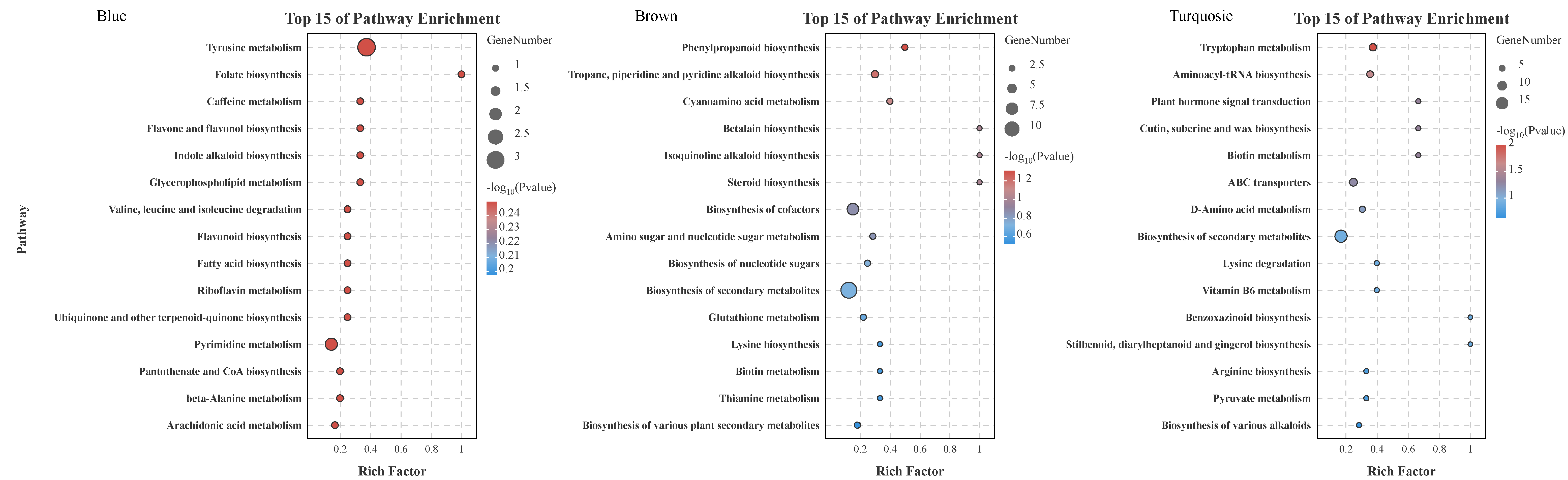

Supplement: Supplementary file 1 [file foods-15-01299-s001.zip › Figure S3.tif]
